# 10α-Hy­droxy-4,9-dimethyl-13-(pipyridin-1-ylmethyl)-3,8,15-trioxatetra­cyclo­[10.3.0.0^2,4^.0^7,9^]tetra­decan-14-one

**DOI:** 10.1107/S1600536811042644

**Published:** 2011-10-22

**Authors:** Mohamed Moumou, Ahmed Benharref, Abdelghani Oudahmane, Ahmed Elhakmaoui, Moha Berraho

**Affiliations:** a Laboratoire de Chimie Biomoléculaire, Substances Naturelles et Réactivité, URAC 16, Faculté des Sciences Semlalia, BP 2390, Bd My Abdellah, 40000 Marrakech, Morocco; b Universite Blaise Pascal, Laboratoire des Materiaux, Inorganiques, UMR CNRS 6002, 24 Avenue des Landais, 63177 Aubière, France; c Laboratoire de Chimie Bioorganique et Analytique, URAC 22, BP 146, FSTM, Université Hassan II, Mohammedia-Casablanca 20810 Mohammedia, Morocco

## Abstract

The title compound, C_20_H_31_NO_5_, was synthesized from 9α-hy­droxy­parthenolide (9α-hy­droxy-4,8-dimethyl-12-methylen-3,14-dioxa-tricyclo­[9.3.0.0^2^,^4^]tetra­dec-7-en-13-one), which was isolated from the chloro­form extract of the aerial parts of *Anvillea radiata*. The mol­ecule is built up from fused five-and ten-membered rings with the pipyridin-1-yl-methyl group as a substituent. The ten-membered ring adopts an approximate chair–chair conformation, while the six- and five-membered rings display chair and envelope conformations, respectively. The dihedral angle between the mean planes of the ten-membered ring and the lactone ring is 20.8 (3)°. An intra­molecular O—H⋯N hydrogen-bond occurs. The crystal structure is stabilized by weak inter­molecular C—H⋯O hydrogen bonds.

## Related literature

For background to the medicinal uses of the plant *Anvillea radiata*, see: El Hassany *et al.* (2004 ▶); Qureshi *et al.* (1990 ▶). For the reactivity of this sesquiterpene, see: Hwang *et al.* (2006 ▶); Neukirch *et al.* (2003 ▶); Neelakantan *et al.* (2009 ▶); Moumou *et al.* (2010 ▶). For ring puckering parameters, see: Cremer & Pople (1975 ▶). For conformations of ten-membered rings, see: Castaneda-Acosta *et al.* (1997 ▶); Watson & Zabel (1982 ▶).
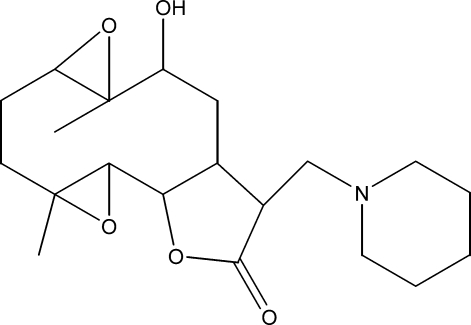


## Experimental

### 

#### Crystal data

C_20_H_31_NO_5_
*M* *_r_* = 365.46
Orthorhombic, 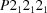

*a* = 8.0899 (5) Å
*b* = 10.7562 (6) Å
*c* = 22.5093 (13) Å
*V* = 1958.7 (2) Å^3^
*Z* = 4
Mo *K*α radiation
μ = 0.09 mm^−1^
*T* = 296 K
0.85 × 0.48 × 0.36 mm

#### Data collection

Bruker X8 APEX CCD area-detector diffractometer
8978 measured reflections
2291 independent reflections
1707 reflections with *I* > 2σ(*I*)
*R*_int_ = 0.036

#### Refinement

*R*[*F* ^2^ > 2σ(*F* ^2^)] = 0.036
*wR*(*F* ^2^) = 0.087
*S* = 1.07
2291 reflections
239 parameters
H-atom parameters constrained
Δρ_max_ = 0.16 e Å^−3^
Δρ_min_ = −0.16 e Å^−3^

### 

Data collection: *APEX2* (Bruker, 2005 ▶); cell refinement: *SAINT* (Bruker, 2005 ▶); data reduction: *SAINT*; program(s) used to solve structure: *SHELXS97* (Sheldrick,2008 ▶); program(s) used to refine structure: *SHELXL97* (Sheldrick,2008 ▶); molecular graphics: *ORTEP-3 for Windows* (Farrugia, 1997 ▶) and *PLATON* (Spek, 2009 ▶); software used to prepare material for publication: *WinGX* publication routines (Farrugia, 1999 ▶).

## Supplementary Material

Crystal structure: contains datablock(s) I, global. DOI: 10.1107/S1600536811042644/fj2458sup1.cif

Structure factors: contains datablock(s) I. DOI: 10.1107/S1600536811042644/fj2458Isup2.hkl

Additional supplementary materials:  crystallographic information; 3D view; checkCIF report

## Figures and Tables

**Table 1:** Hydrogen-bond geometry (Å, °)

*D*—H⋯*A*	*D*—H	H⋯*A*	*D*⋯*A*	*D*—H⋯*A*
O1—H21⋯N1	0.82	2.11	2.927 (2)	174
C1—H1⋯O5^i^	0.98	2.47	3.322 (3)	146
C10—H10⋯O4^ii^	0.98	2.42	3.325 (3)	153

Symmetry codes: (i) 
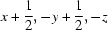
; (ii) 
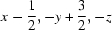
.

**Fig. 1.**
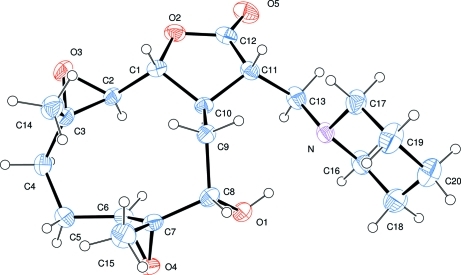
: Molecular structure of the title compound with the atom-labelling scheme. Displacement ellipsoids are drawn at the 30% probability level. H atoms are represented as small spheres of arbitrary radii.

**Fig. 2.**
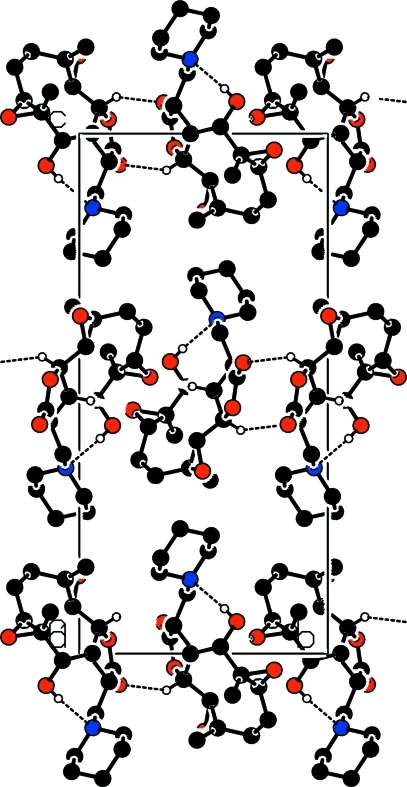
: Packing view showing the C–H···O and O–H···N hydrogen bonds as dashed lines. H atoms not involved in hydrogen bonding have been omitted for clarity.

## supplementary crystallographic information

### Comment

Our work lies within the framework of the valorization of medicinals plants and concerning the Anvillea radiata. The main constituent of the chloroform extract of aerial parts of this plant is 9α - hydroxypartenolide (El Hassany *et al.*, 2004). The reactivity of this sesquiterpene lactone and its derivatives has been the subject of several studies(Neukirch *et al.*, 2003; Hwang *et al.*, 2006; Neelakantan *et al.*, 2009), with the aim to prepare products with a high added value that can be used in the pharmacological industry. In the same context, we have synthesized from 9α-hydroxyparthenolide the 6β,7α-epoxy-9apha hydoxy partenolide (Moumou *et al.*, 2010).This epoxy-hydroxypartenolide treated with one equivalent of pipyridin gives 10α- hydroxy-4,9- dimethyl-13-pipyridin-1-ylmethyl-3,8,15-tioxa-tetracyclo [10. 3. 0.0^2^,^4^.0^7^,^9^]tetradecan-14-one with a yield of 90%. The structure of this new product was determined by its single-crystal X-ray structure. The molecule contains two fused rings which exhibit different conformations with a pyridin ring as a substituent to the lactone ring. The molecular structure of (I), Fig.1, shows the lactone ring to adopt an envelope conformation, as indicated by Cremer & Pople (1975) puckering parameters Q = 0.301 (2)Å and φ = 79.0 (4)°. The ten-membered ring displays an approximate chair-chair conformation, while the pyridin ring has a perfect chair conformation with QT = 0.567 (3) Å, θ = 180.0 (3)° and φ2 = 168 (12)°.This is the typical conformation observed for other sesquiterpenes lactones (Watson & Zabel, 1982; Castaneda-Acosta *et al.*, 1997). In the crystal structure, the molecules are linked by C—H···O intermolecular hydrogen bonds into chains along the *b* axis (Fig.2). In addition an intramolecular O—H···N hydrogen bond is also observed.

### Experimental

The mixture of 6β,7α-epoxy-9apha hydoxy partenolide (0.5 g, 2 mmol) and one equivalent of pipyridin in EtOH (20 ml) was stirred for one night at room temperature. The next day the reaction was stopped by adding water (10 ml) and extracted three times with ethyl acetate (3 *x* 20 ml). The combined organic layers were dried over anhydrous MgSO_4_, filtered and concentrated under vacuum to give 657 mg (1.8 mmol) of 10α- hydroxy-4,9- dimethyl-13-pipyridin-1-ylmethyl-3,8,15-tioxa-tetracyclo [10. 3. 0.0^2^,^4^.0^7^,^9^]tetradecan-14-one, a white solid which was recrystallized in ethyl acetate.

### Refinement

All H atoms were fixed geometrically and treated as riding with C—H = 0.96 Å (methyl), 0.97 Å (methylene), 0. 98Å (methine) with *U*_iso_(H) = 1.2*U*_eq_ (methylene, methine) or *U*_iso_(H) = 1.5*U*_eq_ (methyl, OH). In the absence of significant anomalous scattering, the absolute configuration could not be reliably determined and thus 1692 Friedel pairs were merged and any references to the Flack parameter were removed.

### Crystal data

**Table:**

C_20_H_31_NO_5_	*F*(000) = 792
*M**_r_* = 365.46	*D*_x_ = 1.236 Mg m^−^^3^
Orthorhombic, *P*2_1_2_1_2_1_	Mo *K*α radiation, λ = 0.71073 Å
Hall symbol: P 2ac 2ab	Cell parameters from 8987 reflections
*a* = 8.0899 (5) Å	θ = 2.6–26.4°
*b* = 10.7562 (6) Å	µ = 0.09 mm^−^^1^
*c* = 22.5093 (13) Å	*T* = 296 K
*V* = 1958.7 (2) Å^3^	Prism, colourless
*Z* = 4	0.85 × 0.48 × 0.36 mm

### Data collection

**Table:**

Bruker X8 APEX CCD area-detector diffractometer	1707 reflections with *I* > 2σ(*I*)
Radiation source: fine-focus sealed tube	*R*_int_ = 0.036
graphite	θ_max_ = 26.4°, θ_min_ = 3.3°
φ and ω scans	*h* = −10→6
8978 measured reflections	*k* = −13→13
2291 independent reflections	*l* = −21→28

### Refinement

**Table:**

Refinement on *F*^2^	Secondary atom site location: difference Fourier map
Least-squares matrix: full	Hydrogen site location: inferred from neighbouring sites
*R*[*F*^2^ > 2σ(*F*^2^)] = 0.036	H-atom parameters constrained
*wR*(*F*^2^) = 0.087	*w* = 1/[σ^2^(*F*_o_^2^) + (0.0377*P*)^2^ + 0.1661*P*] where *P* = (*F*_o_^2^ + 2*F*_c_^2^)/3
*S* = 1.07	(Δ/σ)_max_ < 0.001
2291 reflections	Δρ_max_ = 0.16 e Å^−^^3^
239 parameters	Δρ_min_ = −0.16 e Å^−^^3^
0 restraints	Extinction correction: *SHELXL*, Fc^*^=kFc[1+0.001xFc^2^λ^3^/sin(2θ)]^-1/4^
Primary atom site location: structure-invariant direct methods	Extinction coefficient: 0.035 (3)

### Special details

**Table:**

Geometry. All s.u.'s (except the s.u. in the dihedral angle between two l.s. planes) are estimated using the full covariance matrix. The cell s.u.'s are taken into account individually in the estimation of s.u.'s in distances, angles and torsion angles; correlations between s.u.'s in cell parameters are only used when they are defined by crystal symmetry. An approximate (isotropic) treatment of cell s.u.'s is used for estimating s.u.'s involving l.s. planes.

### Fractional atomic coordinates and isotropic or equivalent isotropic displacement parameters (Å^2^)

**Table:**

	*x*	*y*	*z*	*U*_iso_*/*U*_eq_	
C1	−0.2580 (3)	0.4198 (2)	−0.04973 (10)	0.0372 (6)	
H1	−0.2042	0.3507	−0.0705	0.045*	
C2	−0.2861 (3)	0.5264 (2)	−0.09033 (10)	0.0368 (6)	
H2	−0.3339	0.5994	−0.0707	0.044*	
C3	−0.1874 (3)	0.5566 (2)	−0.14313 (10)	0.0413 (6)	
C4	−0.1764 (4)	0.6933 (2)	−0.15778 (11)	0.0512 (7)	
H4A	−0.2786	0.7333	−0.1459	0.061*	
H4B	−0.1655	0.7026	−0.2005	0.061*	
C5	−0.0314 (4)	0.7599 (2)	−0.12752 (11)	0.0521 (7)	
H5A	0.0689	0.7426	−0.1497	0.062*	
H5B	−0.0505	0.8488	−0.1291	0.062*	
C6	−0.0053 (3)	0.7223 (2)	−0.06357 (10)	0.0395 (6)	
H6	−0.1072	0.7104	−0.0408	0.047*	
C7	0.1382 (3)	0.6516 (2)	−0.04136 (10)	0.0389 (6)	
C8	0.1253 (3)	0.5682 (2)	0.01315 (10)	0.0383 (6)	
H8	0.2380	0.5444	0.0245	0.046*	
C9	0.0296 (3)	0.4476 (2)	−0.00041 (10)	0.0360 (5)	
H9A	0.0557	0.4216	−0.0406	0.043*	
H9B	0.0682	0.3830	0.0263	0.043*	
C10	−0.1594 (3)	0.45804 (19)	0.00554 (9)	0.0336 (5)	
H10	−0.1854	0.5453	0.0138	0.040*	
C11	−0.2408 (3)	0.3801 (2)	0.05391 (10)	0.0402 (6)	
H11	−0.1878	0.2981	0.0548	0.048*	
C12	−0.4147 (3)	0.3652 (2)	0.03188 (12)	0.0475 (7)	
C13	−0.2395 (4)	0.4343 (2)	0.11655 (11)	0.0496 (7)	
H13A	−0.2902	0.5160	0.1155	0.060*	
H13B	−0.3068	0.3821	0.1420	0.060*	
C14	−0.0526 (4)	0.4736 (3)	−0.16635 (11)	0.0553 (7)	
H14A	−0.0692	0.3906	−0.1518	0.083*	
H14B	0.0527	0.5039	−0.1531	0.083*	
H14C	−0.0554	0.4732	−0.2090	0.083*	
C15	0.2820 (3)	0.6167 (3)	−0.08001 (12)	0.0577 (8)	
H15A	0.2761	0.6619	−0.1167	0.087*	
H15B	0.2787	0.5291	−0.0881	0.087*	
H15C	0.3833	0.6368	−0.0600	0.087*	
C16	−0.0852 (4)	0.5186 (2)	0.19775 (11)	0.0609 (8)	
H16A	−0.1613	0.4786	0.2250	0.073*	
H16B	−0.1281	0.6007	0.1888	0.073*	
C17	−0.0073 (4)	0.3219 (2)	0.15623 (12)	0.0555 (7)	
H17A	−0.0010	0.2739	0.1198	0.067*	
H17B	−0.0819	0.2791	0.1830	0.067*	
C18	0.0815 (5)	0.5311 (3)	0.22704 (13)	0.0763 (11)	
H18A	0.0696	0.5773	0.2638	0.092*	
H18B	0.1548	0.5776	0.2012	0.092*	
C19	0.1611 (4)	0.3277 (3)	0.18398 (13)	0.0736 (10)	
H19A	0.2387	0.3637	0.1560	0.088*	
H19B	0.1983	0.2443	0.1934	0.088*	
C20	0.1572 (5)	0.4055 (3)	0.24020 (13)	0.0764 (10)	
H20A	0.0927	0.3633	0.2704	0.092*	
H20B	0.2687	0.4164	0.2552	0.092*	
N1	−0.0753 (3)	0.44545 (17)	0.14266 (8)	0.0434 (5)	
O1	0.0559 (2)	0.63372 (15)	0.06146 (7)	0.0471 (5)	
H21	0.0226	0.5841	0.0864	0.071*	
O2	−0.4209 (2)	0.38309 (15)	−0.02731 (8)	0.0470 (5)	
O3	−0.3509 (2)	0.50418 (16)	−0.14908 (7)	0.0496 (5)	
O4	0.1255 (2)	0.78432 (15)	−0.03166 (7)	0.0490 (5)	
O5	−0.5382 (3)	0.34028 (19)	0.05929 (9)	0.0674 (6)	

### Atomic displacement parameters (Å^2^)

**Table:**

	*U*^11^	*U*^22^	*U*^33^	*U*^12^	*U*^13^	*U*^23^
C1	0.0344 (13)	0.0347 (11)	0.0426 (13)	−0.0018 (11)	−0.0010 (11)	−0.0057 (10)
C2	0.0345 (14)	0.0383 (12)	0.0375 (12)	−0.0001 (11)	−0.0080 (10)	−0.0038 (10)
C3	0.0428 (15)	0.0471 (13)	0.0340 (12)	−0.0030 (13)	−0.0071 (11)	−0.0045 (11)
C4	0.0597 (18)	0.0551 (15)	0.0389 (14)	−0.0029 (15)	−0.0084 (13)	0.0070 (12)
C5	0.0610 (18)	0.0456 (14)	0.0496 (15)	−0.0062 (14)	−0.0019 (14)	0.0091 (12)
C6	0.0409 (14)	0.0355 (11)	0.0420 (13)	−0.0074 (12)	−0.0008 (11)	−0.0004 (10)
C7	0.0333 (13)	0.0410 (12)	0.0425 (13)	−0.0068 (12)	−0.0001 (11)	−0.0077 (11)
C8	0.0322 (13)	0.0428 (12)	0.0399 (12)	0.0027 (12)	−0.0059 (10)	−0.0066 (11)
C9	0.0378 (13)	0.0321 (11)	0.0382 (12)	0.0045 (11)	−0.0044 (11)	−0.0013 (10)
C10	0.0364 (13)	0.0266 (10)	0.0378 (12)	0.0022 (11)	−0.0010 (10)	−0.0032 (10)
C11	0.0468 (16)	0.0319 (11)	0.0421 (13)	0.0018 (12)	0.0052 (12)	0.0025 (10)
C12	0.0490 (17)	0.0343 (12)	0.0592 (17)	−0.0048 (13)	0.0064 (15)	0.0018 (12)
C13	0.0587 (18)	0.0461 (13)	0.0440 (14)	0.0098 (15)	0.0126 (13)	0.0034 (12)
C14	0.0554 (18)	0.0632 (16)	0.0472 (15)	−0.0023 (16)	0.0081 (13)	−0.0105 (13)
C15	0.0395 (16)	0.0745 (18)	0.0592 (17)	−0.0019 (16)	0.0072 (13)	−0.0018 (15)
C16	0.101 (3)	0.0442 (14)	0.0379 (14)	0.0050 (17)	0.0115 (16)	−0.0063 (12)
C17	0.074 (2)	0.0468 (14)	0.0460 (14)	0.0131 (15)	−0.0078 (15)	−0.0031 (13)
C18	0.116 (3)	0.070 (2)	0.0428 (15)	−0.024 (2)	0.0013 (19)	−0.0148 (15)
C19	0.081 (2)	0.084 (2)	0.0565 (18)	0.019 (2)	−0.0138 (18)	−0.0114 (16)
C20	0.086 (2)	0.091 (2)	0.0522 (18)	−0.001 (2)	−0.0110 (17)	−0.0086 (16)
N1	0.0642 (15)	0.0354 (10)	0.0305 (10)	0.0038 (11)	0.0050 (10)	−0.0030 (9)
O1	0.0609 (12)	0.0430 (9)	0.0374 (9)	−0.0072 (10)	0.0007 (9)	−0.0079 (7)
O2	0.0399 (10)	0.0453 (9)	0.0558 (11)	−0.0112 (9)	−0.0041 (9)	0.0024 (8)
O3	0.0468 (11)	0.0607 (11)	0.0414 (9)	−0.0071 (10)	−0.0148 (8)	−0.0030 (8)
O4	0.0540 (11)	0.0392 (8)	0.0538 (10)	−0.0127 (9)	−0.0059 (9)	−0.0042 (8)
O5	0.0545 (13)	0.0677 (12)	0.0801 (14)	−0.0161 (11)	0.0205 (12)	0.0067 (11)

### Geometric parameters (Å, °)

**Table:**

C1—O2	1.465 (3)	C11—C13	1.526 (3)
C1—C2	1.484 (3)	C11—H11	0.9800
C1—C10	1.534 (3)	C12—O5	1.204 (3)
C1—H1	0.9800	C12—O2	1.347 (3)
C2—O3	1.442 (3)	C13—N1	1.457 (3)
C2—C3	1.468 (3)	C13—H13A	0.9700
C2—H2	0.9800	C13—H13B	0.9700
C3—O3	1.445 (3)	C14—H14A	0.9600
C3—C14	1.503 (4)	C14—H14B	0.9600
C3—C4	1.509 (3)	C14—H14C	0.9600
C4—C5	1.534 (4)	C15—H15A	0.9600
C4—H4A	0.9700	C15—H15B	0.9600
C4—H4B	0.9700	C15—H15C	0.9600
C5—C6	1.510 (3)	C16—N1	1.471 (3)
C5—H5A	0.9700	C16—C18	1.507 (5)
C5—H5B	0.9700	C16—H16A	0.9700
C6—O4	1.442 (3)	C16—H16B	0.9700
C6—C7	1.475 (3)	C17—N1	1.470 (3)
C6—H6	0.9800	C17—C19	1.500 (4)
C7—O4	1.448 (3)	C17—H17A	0.9700
C7—C15	1.500 (3)	C17—H17B	0.9700
C7—C8	1.524 (3)	C18—C20	1.512 (5)
C8—O1	1.413 (3)	C18—H18A	0.9700
C8—C9	1.541 (3)	C18—H18B	0.9700
C8—H8	0.9800	C19—C20	1.518 (4)
C9—C10	1.540 (3)	C19—H19A	0.9700
C9—H9A	0.9700	C19—H19B	0.9700
C9—H9B	0.9700	C20—H20A	0.9700
C10—C11	1.524 (3)	C20—H20B	0.9700
C10—H10	0.9800	O1—H21	0.8200
C11—C12	1.501 (4)		
			
O2—C1—C2	106.41 (19)	C10—C11—C13	116.5 (2)
O2—C1—C10	105.09 (17)	C12—C11—H11	108.7
C2—C1—C10	111.82 (18)	C10—C11—H11	108.7
O2—C1—H1	111.1	C13—C11—H11	108.7
C2—C1—H1	111.1	O5—C12—O2	120.5 (3)
C10—C1—H1	111.1	O5—C12—C11	129.2 (3)
O3—C2—C3	59.51 (14)	O2—C12—C11	110.3 (2)
O3—C2—C1	119.48 (19)	N1—C13—C11	114.2 (2)
C3—C2—C1	125.9 (2)	N1—C13—H13A	108.7
O3—C2—H2	113.7	C11—C13—H13A	108.7
C3—C2—H2	113.7	N1—C13—H13B	108.7
C1—C2—H2	113.7	C11—C13—H13B	108.7
O3—C3—C2	59.36 (15)	H13A—C13—H13B	107.6
O3—C3—C14	113.6 (2)	C3—C14—H14A	109.5
C2—C3—C14	123.0 (2)	C3—C14—H14B	109.5
O3—C3—C4	114.5 (2)	H14A—C14—H14B	109.5
C2—C3—C4	115.1 (2)	C3—C14—H14C	109.5
C14—C3—C4	117.4 (2)	H14A—C14—H14C	109.5
C3—C4—C5	113.8 (2)	H14B—C14—H14C	109.5
C3—C4—H4A	108.8	C7—C15—H15A	109.5
C5—C4—H4A	108.8	C7—C15—H15B	109.5
C3—C4—H4B	108.8	H15A—C15—H15B	109.5
C5—C4—H4B	108.8	C7—C15—H15C	109.5
H4A—C4—H4B	107.7	H15A—C15—H15C	109.5
C6—C5—C4	113.9 (2)	H15B—C15—H15C	109.5
C6—C5—H5A	108.8	N1—C16—C18	111.6 (2)
C4—C5—H5A	108.8	N1—C16—H16A	109.3
C6—C5—H5B	108.8	C18—C16—H16A	109.3
C4—C5—H5B	108.8	N1—C16—H16B	109.3
H5A—C5—H5B	107.7	C18—C16—H16B	109.3
O4—C6—C7	59.51 (14)	H16A—C16—H16B	108.0
O4—C6—C5	117.0 (2)	N1—C17—C19	112.9 (2)
C7—C6—C5	124.8 (2)	N1—C17—H17A	109.0
O4—C6—H6	114.6	C19—C17—H17A	109.0
C7—C6—H6	114.6	N1—C17—H17B	109.0
C5—C6—H6	114.6	C19—C17—H17B	109.0
O4—C7—C6	59.12 (14)	H17A—C17—H17B	107.8
O4—C7—C15	112.9 (2)	C16—C18—C20	111.6 (3)
C6—C7—C15	122.9 (2)	C16—C18—H18A	109.3
O4—C7—C8	117.02 (18)	C20—C18—H18A	109.3
C6—C7—C8	121.5 (2)	C16—C18—H18B	109.3
C15—C7—C8	111.9 (2)	C20—C18—H18B	109.3
O1—C8—C7	110.69 (18)	H18A—C18—H18B	108.0
O1—C8—C9	111.89 (19)	C17—C19—C20	110.6 (3)
C7—C8—C9	111.75 (18)	C17—C19—H19A	109.5
O1—C8—H8	107.4	C20—C19—H19A	109.5
C7—C8—H8	107.4	C17—C19—H19B	109.5
C9—C8—H8	107.4	C20—C19—H19B	109.5
C10—C9—C8	114.86 (18)	H19A—C19—H19B	108.1
C10—C9—H9A	108.6	C18—C20—C19	109.7 (2)
C8—C9—H9A	108.6	C18—C20—H20A	109.7
C10—C9—H9B	108.6	C19—C20—H20A	109.7
C8—C9—H9B	108.6	C18—C20—H20B	109.7
H9A—C9—H9B	107.5	C19—C20—H20B	109.7
C11—C10—C1	101.97 (17)	H20A—C20—H20B	108.2
C11—C10—C9	116.8 (2)	C13—N1—C17	110.5 (2)
C1—C10—C9	115.23 (19)	C13—N1—C16	109.5 (2)
C11—C10—H10	107.4	C17—N1—C16	109.18 (19)
C1—C10—H10	107.4	C8—O1—H21	109.5
C9—C10—H10	107.4	C12—O2—C1	110.24 (19)
C12—C11—C10	103.16 (19)	C2—O3—C3	61.13 (14)
C12—C11—C13	110.6 (2)	C6—O4—C7	61.38 (15)

### Hydrogen-bond geometry (Å, °)

**Table:**

*D*—H···*A*	*D*—H	H···*A*	*D*···*A*	*D*—H···*A*
O1—H21···N1	0.82	2.11	2.927 (2)	174
C1—H1···O5^i^	0.98	2.47	3.322 (3)	146
C10—H10···O4^ii^	0.98	2.42	3.325 (3)	153

Symmetry codes:  (i) *x*+1/2, −*y*+1/2, −*z*; (ii) *x*−1/2, −*y*+3/2, −*z*.
